# Enhancing of Osseointegration with Propolis-Loaded TiO_2_ Nanotubes in Rat Mandible for Dental Implants

**DOI:** 10.3390/ma11010061

**Published:** 2018-01-01

**Authors:** Nithideth Somsanith, Yu-Kyoung Kim, Young-Seok Jang, Young-Hee Lee, Ho-Keun Yi, Jong-Hwa Jang, Kyoung-A Kim, Tae-Sung Bae, Min-Ho Lee

**Affiliations:** 1 Department of Dental Biomaterials, Institute of Biodegradable Materials, BK21 plus Program, School of Dentistry, Chonbuk National University, JeonJu 54896, Korea; nithideth@hotmail.com (N.S.); yk0830@naver.com (Y.-K.K.); pooh3180@hotmail.com (Y.-S.J.); bts@jbnu.ac.kr (T.-S.B.); 2 Department of Prosthodontics, University of Health Sciences, Vientiane 7444, Laos; 3 Department of Molecular Biology and the Institute for Molecular biology and Gemetics, Chonbuk National University, JeonJu 54896, Korea; leeyh@jbnu.ac.kr; 4 Department of Oral Biochemistry, Institute of Oral Bioscience, BK21 plus Program, School of Dentistry, Chonbuk National University, JeonJu 54896, Korea; yihokn@chonbuk.ac.kr; 5 Department of Dental Hygiene, Hanseo University, Seosan 31962, Korea; jhjang@hanseo.ac.kr; 6 Department of Oral and Maxillofacial Radiology, School of Dentistry and Institute of Oral Bio Science, Chonbuk National University, JeonJu 54896, Korea; beam@jbnu.ac.kr

**Keywords:** dental implant, nanotubes, propolis, mandibular injuries, osseointegration

## Abstract

TiO_2_ nanotubes (TNT) formation is beneficial for improving bone cell–material interaction and drug delivery for Ti dental implants. Among the natural drugs to be installed in TNT, selected propolis has antibacterial and anti-inflammatory properties. It is a resinous natural product which is collected by the honeybees from the various types of plants with their salivary enzymes. This study concludes that TNT loaded with a propolis (PL-TNT-Ti) dental implant has the ability to improve osseointegration. The propolis particles were embedded within the TNT or adhered to the top. In a cytotoxicity test using osteoblast, PL-TNT-Ti group exhibited an increased cell proliferation and differentiation. A Sprague Dawley rat mandibular model was used to evaluate the osseointegration and bone bonding of TNT or PL-TNT-Ti. From the µ-CT and hematoxylin and eosin (HE) histological results after implantation at 1 and 4 weeks to rat mandibular, an increase in the extent of new bone formation and mineral density around the PL-TNT-Ti implant was confirmed. The Masson’s trichrome staining showed the expression of well-formed collagenous for bone formation on the PL-TNT-Ti. Immunohistochemistry staining indicate that bone morphogenetic proteins (BMP-2 and BMP-7) around the PL-TNT-Ti increased the expression of collagen fibers and of osteogenic differentiation whereas the expression of inflammatory cytokine such as interleukin-1 beta (IL-1ß) and tumor necrosis factor-alpha (TNF-α) is decreased.

## 1. Introduction

A successful dental implant treatment requires stability of the implant fixture with the living bone, which could be achieved through the process of osseointegration [1]. Osseointegration is defined as the direct adherence of the bone to the implant surface without any intervening connective tissue [2]. To ensure a direct bone-to-implant contact, it is necessary to shorten the healing time after implantation. Titanium (Ti) is widely used as dental implant materials owing to its low density, good biocompatibility, high corrosion resistance and appropriate mechanical properties [3]. Nevertheless, its bioinert nature warrants more time to achieve complete osseointegration [4]. To overcome this limitation, various physical, chemical, and biochemical treatments are explored to modify the surface of the Ti implant [5,6,7,8]. 

Anodic oxidation, commonly referred as anodizing, is a simple, versatile and cost-effective surface treatment process to generate nanostructures on Ti and its alloys [9]. It involves electrochemically induced dissolution of titanium along with the formation of oxides of titanium. Depending on the type of electrolytes and treatment conditions, the resultant oxide could be a compact layer or porous layer or in the form of nanotubes [10,11]. Recent studies on the interaction between bone cells and TiO_2_ nanotubes (TNT) have shown that osteoblast adhesion, proliferation and migration are significantly influenced by the nanotube structure [12]. The TiO_2_ nanotubes generated by anodizing could promote interaction with osteoblasts by enhancing the bone cell–implant contact area [13]. Furthermore, the nanotube structure has demonstrated benefits for drug delivery and selective release of biomolecules from the implant surface to the surrounding bone as a means of achieving successful dental implantation [14]. One of the major barriers faced after implantation is the possibility of inflammation and its subsequent effects on wound healing and bone bonding. In the expression of macrophages due to external stimuli such as implantation, gram-negative bacteria stimulate the release of lipopolysaccharide (LPS), which is counteracted by the host’s immune cells forcing the release of interferon gamma (IFN-γ). In this process, the LPS activates the macrophages resulting in the release of large amounts of reactive oxygen, nitric oxide, and various cytokines [15]. Hence, it is imperative to prevent the acute or chronic inflammatory responses after implantation so that a better healing around the implant as well as improved osseointegration could be achieved [16].

Propolis is a resinous natural product, which contains a mixture of different plant exudates collected by the honeybees from the buds and leaves of various types of trees and plants and the honeybee’s salivary enzymes [17]. Its composition is quite complex and the main components of propolis been identified as flavonoids and phenolic esters as caffeic acid phenethyl ester [18,19]. The biological activity of propolis primarily depends on the compounds from the polyphenolic fractions, particularly the flavonoids, followed by aromatic acids, phenolic acid esters, etc. [18]. The flavonoids isolated from propolis exhibit anti-microbial and antiprotozoal [20], anti-inflammatory [21], and immunomodulatory [22] activities. The use of propolis is also explored in dental treatment which includes aphthous ulcers, candidiasis, gingivitis, periodontitis and pulpitis [23,24]. Earlier studies using animal models have shown a significant reduction of acute and chronic inflammation with Ethanolic Extract of Propolis (EEP) [25,26,27,28,29]. Hence, it is believed that surface modification of Ti based dental implants with propolis might reduce implant-induced inflammations but also enhance osseointegration. Since the use of TNT formed on dental implants has already been established to display better bone cell–material interactions and drug delivery, the present study aims to load propolis on TNT as a drug-delivery system. For in vitro studies the TNT were prepared on Ti plates whereas for in vivo studies using a rat model, the TNT were prepared on a Ti dental implant. Both were loaded with propolis to study the effect of propolis on osseointegration.

## 2. Results

**Figure 1:**
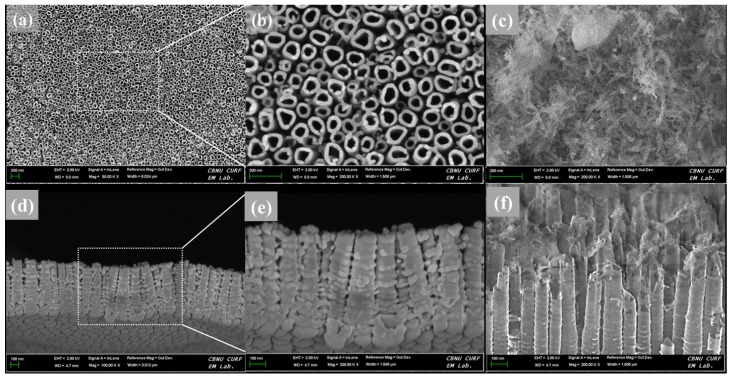
(**a**,**b**) surface and (**d**,**e**) cross sectional morphologies of the TNT formed on Ti plate, and (**c**) surface and (**f**) cross sectional morphologies of the propolis coating on TNT.

The surface and cross-sectional morphologies of the TNT plate after anodization at 20 V for 1 h is shown in Figure 1. The surface morphology reveals a homogeneous distribution of TNT (Figure 1a,b) covering the entire surface of the CP-Ti plate. The formation mechanism of TNT has been reported previously [30,31] and hence not elaborated here. The morphology of the TNT assessed at the cross-section indicates the formation of tubular structures with an open and enlarged shape that contains multiple empty volumes (Figure 1d,e). The morphological features of the TNT suggest that they are ideally suited for drug loading. After propolis load to TNT, the needle–like particles were formed over the surface, and the underlying nanotubes are not visible (Figure 1c). In the cross-sectional image of PL-TNT-Ti (Figure 1f), the particles of propolis located on the nanotubes, and some particles were perpendicular into the nanotube hole or the space between the tubes. But the top part of the nanotube was broken during load the propolis.

**Figure 2:**
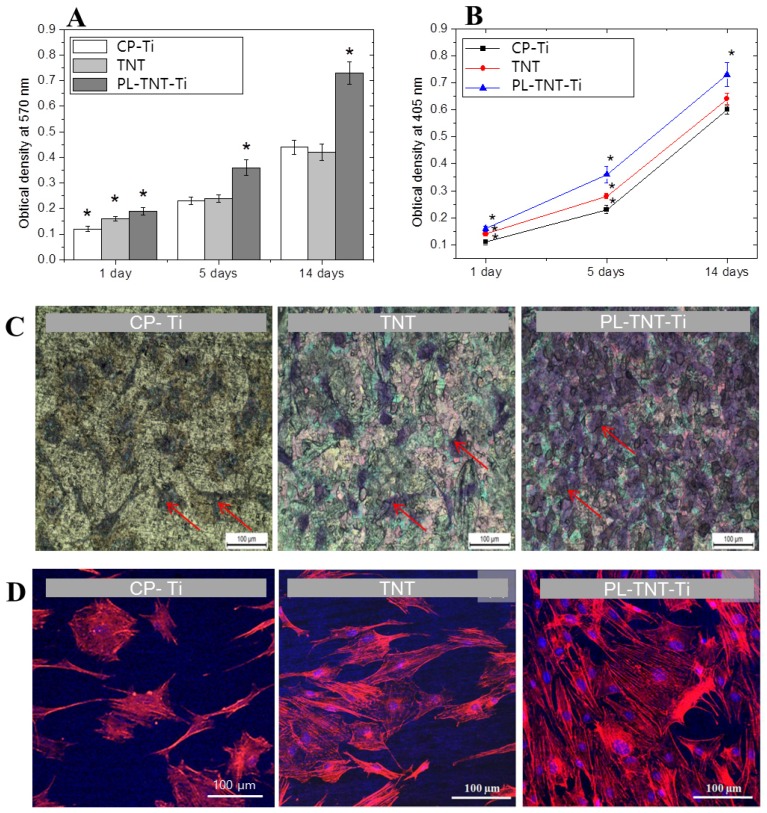
Cell viability of the MC-3T3-E1 cells seeded on CP-Ti, TNT-Ti and PL-TNT-Ti plate seeded with 2 × 10^5^ MC-3T3-E1 cells after 1, 5 and 14 days of cell culture: (**A**) MTT assay; and (**B**) ALP assay (* *p* < 0.05), (**C**) morphology of the cultured cells (red arrow) after 48 h, and (**D**) fluorescence microscopy images of the osteoblast cells after double staining with Rhodmine-phalloidin (red) for actin filaments and 4',6-diamidino-2-phenylindole (DAPI) for nuclei (blue) after incubation for 1 day.

The ability of TNT and PL-TNT-Ti to promote cell proliferation and growth when compared to the CP-Ti was evaluated by a soluble tetrazolium salt [3-(4,5-dimethylthiazol-2-yl)- 2,5-diphenyltetrazolium bromide] (MTT) and alkaline phosphatase (ALP) assays. In the MTT assay, the extent of cell proliferation was estimated by measuring the optical density at 570 nm after culturing the MC3T3-E1 cells on these samples for 1, 5, and 14 days (Figure 2A). After 1 day, the difference in cell viability among the three groups was statistically significant different (*p* = 0.000906). The extent of cell proliferation on the TNT surface did not differ significantly when compared to that occurring on CP-Ti after 5 (*p* = 0.8385) and 14 days (*p* = 0.6499) of culturing. However, the difference became significant when the effect of TNT and PL-TNT-Ti were compared (*p* = 0.00028) after 14 days. 

In the ALP assay, the degree of cell differentiation was estimated by measuring the optical density at 405 nm after culturing the cells on these samples for 1, 5, and 14 days (Figure 2B). It is evident that degree of cell differentiation is much higher on PL-TNT-Ti, followed by TNT and CP-Ti. The trend observed in ALP assay is quite similar to the one inferred from the MTT assay. The degree of cell differentiation after culturing for 1 day showed no significant difference in ALP activity among all three groups (*p* = 0.00135). Moreover, no significant difference in ALP activity of CP-Ti and TNT-Ti could be observed after 5 (*p* = 0.0585) and 14 days (*p* = 0.0651) of cell culturing. In contrast, a significant difference in cell differentiation could be observed for PL-TNT-Ti after 1, 5 and 14 days of cell culture when compared with the other two groups. Figure 2C shows the morphology of the cultured cells by means of crystal violet staining assay. The surface of the PL-TNT-Ti exhibits higher cell viability when compared to TNT and CP-Ti. This trend is further confirmed by the fluorescence images (Figure 2D).

**Figure 3:**
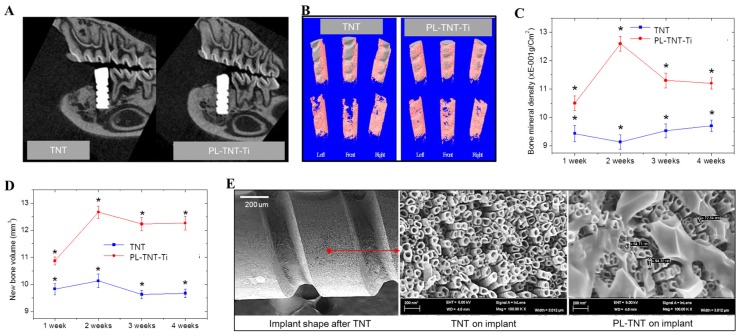
The microcomputed tomographic (µ-CT) analyses of peri-implant tissue at 1, 2, 3 and 4 weeks after implantation at the healed edentulous mandibular site. (**A**) Two-dimensional µ-CT analysis of peri-implant tissue at 4 weeks after implantation; (**B**) Three-dimensional µ-CT analysis of peri-implant tissue at 4 weeks after implantation (the gray color represents the implant whereas the pink and yellow colors represent the bone tissue); (**C**) Three-dimensional µ-CT analysis of bone mineral density around the implant surface; (**D**) Three-dimensional µ-CT analysis of the volume of the new bone formation around the implant surface (* *p* < 0.05); and (**E**) surface morphologies of implant.

For in vivo testing, Ti nanotubes and propolis coated with screw type were coated with uniformed nanotubes with diameters of 60 to 90 nm as plate type in Figure 3E. In the present study, micro-computed tomography (µ-CT) analysis is used to identify the location that is suitable for implantation in rat mandibles and to assess the extent of new bone formation. In addition, the volume of bone mineral density as well as the volume of newly formed bone around the implants is estimated based on µ-CT analysis. It has been established that images acquired in µ-CT analysis are very useful to measure the temporal process of tissue–implant integration under in vivo and to accurately determine the amount of bone formation around implant surfaces [32]. The two-dimensional (2D) µ-CT analysis is used to assess the tooth morphology before and after extraction. Analysis performed at 4 weeks after extraction reveals that the alveolar bone at the implantation site is completely healed. Following this, the TNT and PL-TNT-Ti implants were inserted at the edentulous site of the mandible (Figure 3A). Examination by µ-CT analysis at 1 week after implantation reveals no significant difference in the new bone formation between the TNT and PL-TNT-Ti implants (*p* = 0.00918). However, at 4 weeks after implantation, the 2D µ-CT analysis of the peri-implant tissue image clearly reveals its better anchorage on PL-TNT-Ti implants. This inference is further confirmed by the 3D µ-CT images, which clearly indicate an increased extent of new bone formation on PL-TNT-Ti implant than those formed over TNT implant (Figure 3B,D). In the Figure 3B, the gray color represents the implant shape was shown the gray color, and surrounding the implant in pink represents bone tissue. The volume of the bone mineral density and the volume of newly formed bone around the TNT and PL-TNT-Ti implants are shown in Figure 3C,D and Table 1, respectively. It is evident that the bone mineral density and the volume of newly formed bone around the PL-TNT-Ti implant are much higher than those formed over TNT implant at 1, 2, 3, and 4 weeks after implantation. At 2 weeks after implantation, the bone mineral density around the TNT implant is decreased whereas at a similar time interval, an increased level bone mineral density is observed for PL-TNT-Ti implant (Figure 3C). 

**Figure 4:**
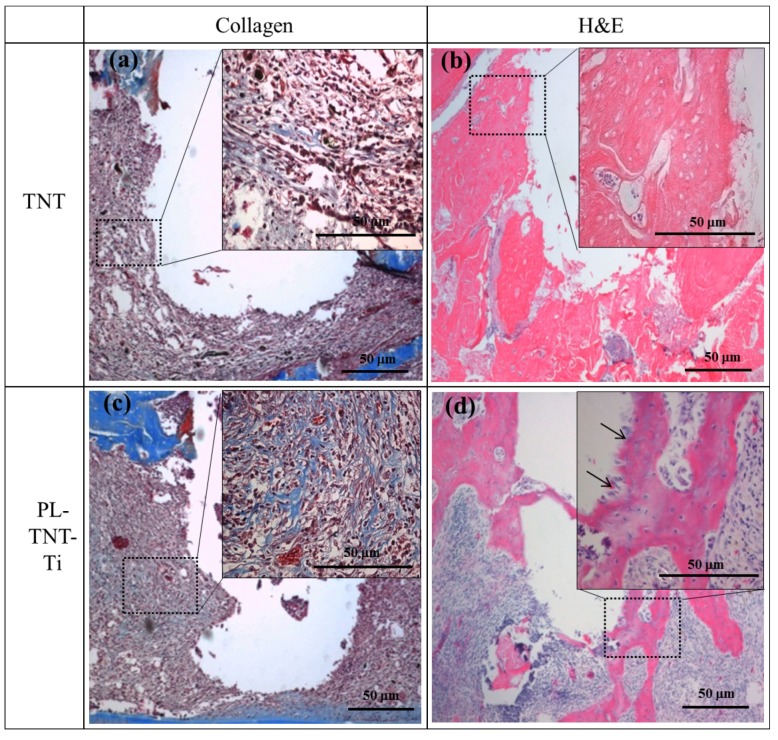
Histological morphologies acquired after Masson’s trichrome and hematoxylin and eosin (HE) staining depicting the expression of collagen and the new bone formation, respectively around the (**a**,**b**) TNT-Ti and (**c**,**d**) PL-TNT-Ti dental implants: (**a**,**c**) Expression of collagen (blue areas); and (**b**,**d**) new bone formation, at 4 weeks after implantation. The arrow marks indicate the osteoblastic rim.

Masson’s trichrome and HE staining were used to evaluate the extent of new bone formation around the surface of TNT and PL-TNT-Ti implants at 4 weeks after implantation. The histological morphologies acquired after Masson’s trichrome and HE staining performed around the surface of TNT and PL-TNT-Ti implants are shown in Figure 4. The Masson’s trichrome staining indicates the formation of only a few collagen fibers around the surface of the TNT implant (Figure 4a). The newly formed bone around this implant surface reveals the presence of abundant fibrotic tissue and immature bone formation with a few osteoblast cells (Figure 4b). In contrast, the Masson’s trichrome staining showed the expression as blue of well-formed collagenous bone trabecules, and muscle fibers and cytoplasm were marked as red (Figure 4c). The formation of new bone with concentration of macrophages and nuclei around the surface of the PL-TNT-Ti implant (Figure 4d).

**Figure 5:**
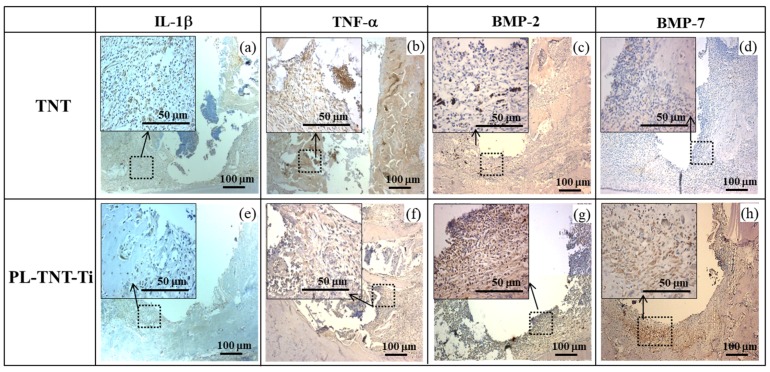
Histological morphologies from IHC staining show the expression of IL-1ß, TNF-α, BMP-2, and BMP-7 around dental implants; the expressions of inflammatory molecules such as IL-1β (**a**,**e**), and TNF-α (**b**,**f**) at 1 week after implantation, the expressions of BMP-2 (**c**,**g**), and BMP-7 (**d**,**h**). The brown color indicates positive cells at 4 weeks after implantation.

The histological morphologies acquired after immunohistochemical (IHC) staining at 1 week as IL-1ß, and TNF-α and at 4 weeks as BMP-2 and BMP-7 after implantation is shown in Figure 5. IL-1ß, and TNF-α positive cells were stained brown, and the majority of these cells which were likely inflammatory cells. A decrease in the expression of inflammatory cytokines such as IL-1ß, and TNF-α is observed around the surface of PL-TNT-Ti implants (Figure 5e,f). Particularly, the expression of inflammatory cytokines (TNF-α) showed the whole tissue around the TNT implant, but partial expression of PL-TNT. The expression of bone formation molecules BMP-2 and 7 of the PL-TNT-Ti implant (Figure 5g,h) as brown color is relatively higher than those observed around the TNT implant (Figure 5c,d). As evidenced by the increased expression of bone formation by BMP-2 and BMP-7 (Figure 5g,h), around the surface of PL-TNT-Ti implant site showed dense bone cell growth.

## 3. Discussion

The mechanism of nanotubular TiO_2_ formation on the Ti is due to a dynamic equilibrium between the electrochemically assisted growth and the simultaneous dissolution of the oxide layer by the fluoride ions in the electrolyte. The chemical reactions of oxide layer forms on titanium can be expressive of 2H_2_O → 4H^+^ + O_2_ + 4e^−^. Due to the presence of fluoride ions, the oxide layer dissolves locally and a nanotube is newly formed from pits parts between TiO_2_ and HF of the oxide layer follow as TiO_2_ + 4H^+^ + 6F^−^ → TiF^2−^_6_ + 2H_2_O. The determination of TiO_2_ nanotube pore size is related to the current density because it changes the electrochemical etching rate [33]. As the current density increases, the electrochemical etching rate and power, and field strength increase, correspondingly increasing the pores and voids of the tube. Since we have previously controlled the pore and length of nanotubes and continued to research related drug loading [34,35,36], the optimal conditions were applied to this study.

In this study, an alcohol-free extract that contains about 60% crude propolis collected by the honeybees mainly from the exudates of poplars [37] and sourced from New Zealand was used. It can be categorized as “European” type propolis, which possesses anti-inflammatory and anticancer properties, mainly due to the presence of major amounts of chrysin and caffeic acid phenethyl ester (CAPE) as its main constituents [38,39]. Propolis possesses a significant anti-inflammatory effect under both chronic and acute inflammation conditions [27]. The biological effect of propolis on the surface of Ti implants has been shown to enhance bone formation around the implant [40]. It is well-known that TNT can be successfully used as an appropriate vehicle for localized drug delivery Propolis is loaded on the TNT implant with a view to increase the extent of bone formation and to promote osseointegration. The ability of the implant surface to promote osteoblastic cell growth and proliferation of the MC3T3-E1 cells is assessed using a MTT assay. Over a period of 5 and 14 days, the MTT assay showed no significant difference between the CP-Ti and the TNT. However, a significant difference in these attributes could be observed when the ability of CP-Ti and the TNT implants is compared with that of the PL-TNT-Ti implant. The osteoblast cells could adhere and spread very well on the surface of the PL-TNT-Ti implant.

Cell viability is much greater over the PL-TNT-Ti plate than on the CP-Ti and the TNT plates. Hence, it is clear that the improvement in cell viability is due to the presence of propolis in the TNT. The increase in optical density with an increase in cell culture time confirms the ability of propolis loaded in the TNT to exhibit good in vitro biocompatibility. Cell proliferation and differentiation over a material measured as a function of time is also good indicator of its bioactivity towards tissue engineering. The results of the present study suggest that the surface of PL-TNT-Ti had a good tendency to increase the cell viability when compared to the CP-Ti and the TNT surfaces. A previous study [41] reported that dielectrophoresis (DEP) showed cytotoxic activity against a PC-3 cancer cell line based on an MTT assay. ALP is an early marker of the osteoblast differentiation and it plays an important role in mineralization [42]. The process of mineralization is accompanied by an increased expression of the osteoblast-associated proteins osteocalcin and ALP [43]. In this study, the PL-TNT-Ti showed much higher level of cell differentiation after culturing for 1, 5 and 14 days. These increased levels of cell differentiation with an increase in cell culture time up to 14 days indicates the beneficial effect of propolis on the mineralization. 

The right mandibular first molar was extracted and used as a model for the in vivo study for 4 weeks to understand the effect of PL-TNT-Ti dental implant. This length of time is typical to allow maximal bone healing preceding implantation. Because titanium has excellent biocompatibility, new bones are formed within a few months after implant placement. Initial inadequate stability results in high implant failure rates, and to achieve its specific drug effect, the healing effect between the implant and the bone at the short term is important [44]. The implants were inserted at the healed edentulous site in order to determine the biological response to CP-Ti, TNT and PL-TNT-Ti dental implants. µ-CT analysis is used to measure the mineralized tissue and the extent of new bone formation. The findings of this study indicate an increase in the extent of new bone formation and mineral density around the PL-TNT-Ti dental implants at 4 weeks after implantation. This inference is supported by the histological observations made using HE staining, which showed new bone formation with regular bone trabeculae around the surface of the PL-TNT-Ti implant. Masson’s trichome staining was also used to detect the expression of collagen secreted in the process of bone formation as an indirect index of the amount of newly formed bone [45]. Collagen, which is synthesized mainly by differentiated osteoblasts, makes up 90% of the organic matrix of bone, constitutes the major protein framework of bone as the template of matrix mineralization, and also sustains the integrity and flexibility of the bone [46]. In this study, Masson’s trichome staining showed the expression of well-formed collagen fibers around the PL-TNT-Ti implants indicating that the surface of these implants enhanced collagen fiber fabrication to form the basic framework of the bone. In contrast, only a few collagen fibers were observed around the TNT implants.

During the healing process, macrophages release large amounts of pro-inflammatory and cytotoxic mediators such as reactive oxygen species and numerous cytokines [15]. Cytokines are polypeptides synthesized in the lymphocytic and monocytic cells, and they play an important role in multiple cellular functions, such as the immunological response, inflammation, and hematopoiesis. Interleukin 1 (IL-1) directly stimulates osteoclastic resorption, increasing the proliferation and differentiation of pre-osteoblasts, as well as the osteoclastic activity, and inhibiting the apoptosis of osteoclasts [47]. Tumor necrosis factor (TNF) is a cytokine involved in inflammation and is a member of a group of cytokines that stimulate the acute phase reaction [48]. Among the pro-inflammatory cytokines, IL-1ß, and TNF-α are the primary mediators of the inflammatory responses, which are related to various chronic inflammatory diseases [49,50]. Thus, in this study, early inflammatory expression as IL-1ß, and TNF-α were assessed for the observation of BMP-2 and BMP-7 expression associated with bone formation.

IHC staining is performed to evaluate the expression of bone formation molecules around the surface of both TNT and PL-TNT-Ti implants at 1 and 4 weeks after implantation. The production of pro-inflammatory cytokines such as IL-1ß, and TNF-α is significantly inhibited on the surface of the PL-TNT-Ti implants. Propolis had significant inhibitory effects on the levels of IL-1β and TNF-α in this rat model, suggesting that one of the possible mechanisms to explain the anti-inflammatory and immune effects of Propolis is its inhibition of the activation and differentiation of mononuclear macrophages. Park et al. [27] have also reported that EEP has anti-inflammatory effects under conditions of both chronic and acute inflammation. According to them, this could be attributed to the inhibitory effect on the formation of prostaglandin. Thus, our results demonstrate that the surface of the PL-TNT-Ti implants promoted new bone formation and enhanced osseointegration around the dental implant. During infection, the innate immune cells liberate inflammatory cytokines such as IL-1ß, and TNF-α, which play an important role in the release of prostanoids [51]. Previous studies suggested that proinflammatory cytokines including TNF-α and IL-1β have trigger to peri-implantities [52,14]. Franchin et al. [53] have studied the antinociceptive activity of the ethanolic extract of geopropolis (EEGP) and its aqueous fractions. According to them, administration of EEGP and its aqueous fraction has led to a reduction in the inflammatory hypernociception, which could be attributed to the inhibition of IL-1β, and TNF-α cytokines and the consequent inhibition of the release of prostanoids, and the interaction between neutrophils and the endothelial cells. It is believed that the phenolic compounds present in propolis could be responsible for this antinoceceptive activity and the exact role of these compounds should be investigated in detail. Orsi et al. [54] has confirmed that the production of IL-1ß, and TNF-α in LPS or LPS + IFN-γ active macrophages is blocked by propolis extracts. Hence, the significant inhibition of pro-inflammatory cytokines as IL-1β and TNF-α on the surface of the PL-TNT-Ti implant observed due to the ability of propolis extract in preventing the inflammatory responses in the present study. It was also evidenced that loaded of propolis on implant might be blocked peri implantitis. 

In osteoblast differentiation, the gene for BMP-2 and 7 is involved in the formation and regeneration of bone. And the equal amounts of BMP-2 and BMP-7 expression vectors induce maximal alkaline phosphatase (ALP) activity in a mouse stromal cell culture system, and induce ectopic bone formation more rapidly than the transfer of single gene [55]. In this study, propolis treatment was significantly higher effect on BMP-2 and 7 expressions. Low inflammatory factors and the expressions of BMP-2 and 7 increased new bone formation around the implant, thus increasing the adhesion with mandibular and implant. 

The reason for the failure of the implant is the fail of inhibit the initial inflammation. Since this study is to observe the effect of the post-implantation on the pathology of the post-implant, the infection of the oral bacteria in the natural state was considered as the same condition as the control. In this study, propolis loading inhibited early inflammation and blocked peri implantitis. In future studies, the mechanism of chemical bonding between propolis and metal should be clarified, and the effect of surface pretreatment of titanium on the attachment of drugs such as propolis should be further investigated.

## 4. Materials and Methods

### 4.1. CP-Ti Preparation and Fabrication of TiO_2_ Nanotubes on CP-Ti Plates

Commercially pure Ti (CP-Ti) plate (99.9% purity, Kobe Steel, Kobe, Japan), cut to a dimension of 10 × 10 × 1 (mm) was used as the substrate material for in vitro studies. They were sequentially polished using a SiC-coated abrasive papers. The mechanically polished CP-Ti samples were pickling in a mixture of 7 wt % HF and 12 wt % HNO_3_, rinsed thoroughly using deionized water to remove the acidic residues, cleaned using deionized water. 

The TiO_2_ nanotubes were fabricated by anodization process as described previously [56]. A solution mixture containing 1 wt % ammonium fluoride, 20 wt % H_2_O and 79 wt % glycerol were used as the electrolyte solution. The CP-Ti sample was used as the anode while a large Pt sheet served as the cathode. Anodization was performed using a direct current constant power supply (DADP-503D, Daunanotek, Gwangmyeong, Korea) at 20 V for 1 h. After anodizing, the samples were rinsed using deionized water and dried. Field emission scanning electron microscopy (S-4700, Hitachi, Tokyo, Japan) was used to confirm the formation of TNT over the surface of the Ti plate after anodization. The TNT formed on CP-Ti plate was used for the in vitro studies.

### 4.2. Loading of Propolis on TNT and Mini Implants for In Vivo Studies

For in vivo studies, pure Ti rod (99.9% purity, Kobe Steel, Kobe, Japan) (length: 4.5 mm; diameter: 0.85 mm) were manufactured mini implants by custom made. A rod with a diameter of 0.85 mm was screw-processed at a thread angle of 20 degrees with a pitch of 0.4 mm and a length of 4.5 mm, and the head was made 2 mm length. When forming the nanotube, the head portion was used to hold the implant to immerse in the electrolyte, and then the head portion was cut off after the surface treatment. It was subjected to anodizing to fabricate TNT on Ti surface. Commercially available propolis (an alcohol-free extract of propolis, MegaMax NZ, Auckland, New Zealand), which contains 60% crude propolis, was used on TNT. The TNT on Ti plates and implants were cleaned with distilled water prior to loading the propolis. The cleaned samples were fully immersed in the propolis solution for 24 h at 25 °C and vacuum-dried at 25 °C for 24 h. The propolis loaded TNT on Ti plate and implant were designated as plate and implant types of PL-TNT-Ti, respectively. 

### 4.3. In Vitro Studies

The untreated CP-Ti, TNT and PL-TNT-Ti plates were used for in vitro studies, which include MTT (3-(4,5-dimethylthiazol-2-yl)-2.5-diphenyl tetrazolium bromide) assay to assess cell proliferation, alkaline phosphatase (ALP) assay to assess cell differentiation and, cell morphology using crystal violet staining and fluorescence microscopy images of osteoblast cells.

MC-3T3-E1 osteoblast-like cells (CRL-2593; American Type Collection, Manassas, VA, USA) in α-MEM media (Gibco BRL, Grand Island, NY, USA) supplemented with 10% fetal bovine serum, 2 mM of glutamine, 100 units/mL of penicillin, and 100 µg/mL of streptomycin, maintained at 37 °C in a humidified 5% CO_2_ atmosphere was used for this study. The MC-3T3-E1 cells were seeded 2 × 10^5^ cells/24 well on CP-Ti, TNT and PL-TNT-Ti plates. The 3-(4,5-dimethylthiazol-2-yl)-2.5-diphenyl tetrazolium bromide (MTT) assay was used to determine the density of cell viability and proliferation after culturing for 1, 5, and 14 days. At the end of the incubation period, the medium of the cultured cells was removed, and the cells were rinsed twice with sterile phosphate buffered saline (PBS). The diluted MTT solution (1000 µg/0.1 mL) was applied to each specimen. After incubating the samples in the MTT solution for 4 h, the solution was removed, and 200 µL of dimethyl sulfoxide (Duksan Pure Chemicals, Ansan, Korea) was applied to each specimen to dissolve the dark-blue crystals of MTT formazan. Optical density (OD) was measured at a wavelength of 540 nm using an ELISA reader (Spectra Max Plus Microplate Reader, Molecular Devices, Sunnyvale, CA, USA).

For crystal violet staining, the cells were then stained with 0.3% crystal violet solution and incubated for 30 min at 25 °C. Cell morphological characteristics were observed using a stereo microscope (SZX7, Olympus, Tokyo, Japan). For measurement of cell adhesion, after 24 h incubation, the osteoblast cells were rinsed with PBS solution, fixed with 4% paraformaldehyde in PBS for 30 min and again washed with PBS. The cells were permeabilized with 0.25% Triton X-100 in PBS (PBST) for 15 min, blocked nonspecific binding with 1% bovine serum albumin in PBST solution for 30 min, stained with Rhodamine-phalloidin for 30 min at 25 °C, washed with PBST. The nuclei of the cells were stained with 0.1 µg·mL^−1^ 4′,6′-diamidino-2-phenylindole (DAPI) in PBST solution for 2 min, and washed with PBST. The cells were visualized using a confocal laser scanning microscopy (LSM700, Carl Zeiss, Munich, Germany).

For Alkaline phosphatase (ALP) assay, 200 µL of lysis buffer solution (10 mM of Tris-HCl, 2 mM magnesium chloride, and 0.1% triton-X-100, pH 7.4) was applied to each well to detect cell lysis after culturing for 1, 5 and 14 days. After performing three freezing thawing cycles (at −80 °C for 2 h followed by treatment at 37 °C for 2 h), 20 µL of the solution was applied to each well of a 96-well microplate, followed by the addition of 100 µl of a 4-nitrophenyl phosphate (pNPP) solution. The microplates were gently shaken and incubated at 37 °C for 30 min. After adding 80 µL of stop buffer (1 M of NaOH), the optical density was measured at a wavelength of 405 nm using an ELISA reader.

### 4.4. Details of the Type of Animals Used and Surgical Procedures Employed

The TNT and PL-TNT-Ti implants were used for in vivo studies, which include micro-computed tomographic (µ-CT) analysis, histological analysis using Masson’s trichrome staining, hematoxylin and eosin (HE), and immunohistochemical (IHC). After TNT and PL-TNT treatment on implants, the surface morphologies were observed with FE-SEM. 

The study was conducted in accordance with the Declaration of Helsinki. The ethical clearance of this study was approved by the Institutional Animal Care and Use Committee of the Chonbuk National University Laboratory Animal Center (CBU 2015-0782). A total of 20 male Sprague-Dawley rats were chosen for the study and they were divided into two groups. All samples and tools for animal test were sterilized by ethylene oxide gas treatment. One group of rats (*n* = 10) were chosen for implantation with TNT implant while the other group (*n* = 10) were used for implanting with PL-TNT-Ti implants for 1 and 4 weeks. The rats were allowed to accommodate well with the environmental conditions (temperature: 25 °C; relative humidity: 30% to 50%) for a week. All surgical procedures were performed under conditions of general anesthesia, induced by intramuscular injection with ketamine (Ketamine HCl, 57.68 mg, Yuhan Corporation, Seoul, Korea) and xylazine hydrochloride (Rompun, Bayer Korea, Seoul, Korea). The first molar of the both side mandible was carefully extracted using an elevator to avoid any possible damage of the extraction socket. Subsequently, the alveolar bone at the site of the extraction was allowed to heal for 1 month. The implant was placed at the healed edentulous site in a cavity of 0.8 mm diameter, created using a drill at 900 rpm. The use of profuse irrigation with saline-solution during drilling operation helped to control damage of the bone tissues from the resultant heat. The Ti screws were carefully inserted in the mandibular bone and tightened using a self-tapping process, until the screw thread had completely entered the cortical bone to provide primary stability to the implant. In order to prevent the pre-infection of the implants, antibiotics (the antibiotic Amikacin sulfate, Samu Median, Yesan, Korea) were injected intramuscularly and the infection control was continued by injecting antibiotics continuously for 3 days after the procedure. 

The change in bone architecture in peri-implant tissue of implantation for 4 weeks was evaluated by micro-computed tomographic (µ-CT) analysis every week. The mandibular bones were scanned using µ-CT (Skyscan, Model 1076, Kontich, Belgium), which was operated at an anode electrical current of 100 kV and 150 µA, with an integration time of 200 ms. The use of an aluminum filter helped to eliminate the hardening beam at a resolution of 18 µm. The mandibular compartments around implants were considered as the main regions of interest (ROI). The volume of interest consisted of the collective sum of all ROI layers over continuous sets of cross-sectional image slices, which represents regenerated bone only. Further, the new bone volume and bone mineral density around the implant were calculated with the use of phantom and Hounsfield units (HU). A bone and implant threshold value in HU by µ-CT was determined to be between low phantom (0.25 g·cm^−3^) 1089.7966 HU and high phantom (0.75 g·cm^−3^) 3241.0643 HU. Since the volume of ROI corresponds to volume of the original bone, the amount of bone present within the ROI is considered to be the newly formed bone. To create 3D images, a binary threshold was selected (gray-scale index: implant area = 160 to 255 µm, new bone area = 100 to 143 µm and total bone area = 70 to 120 µm).

Five rats from each group were sacrificed at one week for immunohistochemical (IHC) staining (tumor necrosis factor-alpha [TNF-α] and interleukin-1 beta [IL-1β]) and 4 weeks for HE, Masson′s stain and IHC staining (BMP-2 and BMP-7) after implantation. The mandibular bones with the implants were isolated and washed with PBS to remove all blood. Bone tissues were fixed in 10% neutral-buffered formalin solution. Decalcification was performed in 15% EDTA and 0.1 M of Tris at pH = 7.0 for 5 weeks to remove the calcified tissues. The flexibility method was used to determine the bone tissues at the endpoint of decalcification. The implants were gently removed, while the mandibular bone tissues were dehydrated in ethanol and cleared in xylene, for embedding in paraffin. The embedded blocks of tissues were cut into 5-µm-thick sections using a manual rotary microtome (Leica RM2235, Leica Biosystems, Germany). Tissue sections were mounted on glass slides and subjected to hematoxylin and eosin (HE) for tissue structure, and the Trichrome (Masson’s) Stain Kit (Sigma-Aldrich. St. Louis, MO, USA) was used to detect the expression of collagen. IHC staining, the former was used to evaluate new bone formation around the implant while the latter was used to detect the expression of bone morphogenetic protein BMP-2, and BMP-7, using the immunohistochemistry accessory kit (Bethyl Laboratories, Montgomery, TX, USA). The primary antibodies of BMP-2 (BMP2 P275 pAb, Bioworld Technology, St. Louis Park, MN, USA) and BMP-7 (BMP7 E173 pAb, Bioworld Technology, St. Louis Park, MN, USA), IL-1β (IL-1β 11E5, Santa Cruz Biotechnology, Inc., Dallas, TX, USA), TNF-α (TNFα C-4, Santa Cruz Biotechnology, Inc., Dallas, TX, USA) and collagen were used at a 1:100 dilution in accordance with the protocol for 30 minutes at room temperature. The stained slides were observed using optical microscopy.

### 4.5. Statistical Analysis

The experimental data of all groups were analyzed independently using one-way analysis of variance (ANOVA) to ascertain the differences in the variables among them. A value of *p* < 0.05 was considered as statistically significant. 

## 5. Conclusions

The effect of the TNT loaded with propolis on the osseointegration of dental implants in the rat mandible is studied. TNT loaded with propolis has increased the osteoblast proliferation and differentiation. New bone formation and bone mineral density were much greater with the propolis-loaded TNT implants when compared to the drug free TNT implants. TNT loaded with propolis decreased the expression of inflammatory cytokines such as IL-1β, and TNF-α, but increased the expression of collagen fibers and osteogenic differentiation proteins, such as BMP-2 and BMP-7. It is important to optimize the surface of dental implants to improve bone formation and osseointegration and TNT loaded with propolis is a viable option to achieve the same.

## Figures and Tables

**Table 1:** The bone mineral density and the volume of the new bone formation around the implant surface from 3D µ-CT analysis.

Period	Bone Mineral Density (xE-001g/cm^2^)				New Bone Volume (mm^3^)			
	TNT		TNT + Propolis		TNT		TNT + Propolis	
	Mean	SD (±)	Mean	SD (±)	Mean	SD (±)	Mean	SD (±)
1 week	9.43	0.292	10.5	0.263	9.83	0.211	10.87	0.150
2 weeks	9.13	0.254	12.6	0.265	10.13	0.252	12.67	0.231
3 weeks	9.53	0.252	11.3	0.261	9.63	0.152	12.23	0.254
4 weeks	9.7	0.201	11.2	0.219	9.67	0.154	12.26	0.251
